# Squamous epitheliotropism of Enterovirus A71 in human epidermis and oral mucosa

**DOI:** 10.1038/srep45069

**Published:** 2017-03-21

**Authors:** Win Kyaw Phyu, Kien Chai Ong, Chee Kwan Kong, Abdul Khalil Alizan, Tindivanam Muthurangam Ramanujam, Kum Thong Wong

**Affiliations:** 1 Department of Pathology, Faculty of Medicine, University of Malaya, Kuala Lumpur, 50603, Malaysia; 2 Department of Biomedical Science, Faculty of Medicine, University of Malaya, Kuala Lumpur, 50603, Malaysia; 3 Department of Surgery, Faculty of Medicine, University of Malaya, Kuala Lumpur, 50603, Malaysia

## Abstract

Hand-foot-and-mouth disease is a self-limiting paediatric infectious disease commonly caused by Enterovirus A71 (Genus: *Enterovirus*, Family: *Picornaviridae*). Typical lesions in and around the hands, feet, oral cavity and other places may rarely be complicated by acute flaccid paralysis and acute encephalomyelitis. Although virus is readily cultured from skin vesicles and oral secretions, the cellular target/s of Enterovirus A71 in human skin and oral mucosa are unknown. In Enterovirus A71-infected human skin and oral mucosa organotypic cultures derived from the prepuce and lip biopsies, focal viral antigens and viral RNA were localized to cytoplasm of epidermal and mucosal squamous cells as early as 2 days post-infection. Viral antigens/RNA were associated with cytoplasmic vacuolation and cellular necrosis. Infected primary prepuce epidermal keratinocyte cultures showed cytopathic effects with concomitant detection of viral antigens from 2 days post-infection. Supernatant and/or tissue homogenates from prepuce skin organotypic cultures and primary prepuce keratinocyte cultures showed viral titres consistent with active viral replication. Our data strongly support Enterovirus A71 squamous epitheliotropism in the human epidermis and oral mucosa, and suggest that these organs are important primary and/or secondary viral replication sites that contribute significantly to oral and cutaneous viral shedding resulting in person-to-person transmission, and viraemia, which could lead to neuroinvasion.

Enterovirus A71 (EV-A71) is a non-enveloped, single-stranded RNA virus which belongs to Enterovirus A^1^ species group of the *Enterovirus* genus in the *Picornaviridae* family^2^. It is one of the most common and important causes of hand-foot-and-mouth disease (HFMD), which is seen mostly in children. Most cases of EV-A71-associated HFMD are mild and self-limited, and typically characterized by ulcerating vesicles and lesions in the oral cavity, hands, feet, and occasionally on the buttocks, knees, and other places^3^,^4^,^5^,^6^,^7^. However EV-A71-associated HFMD may be complicated by aseptic meningitis, acute flaccid paralysis or acute encephalomyelitis^4^,^8^.

Person-to-person EV-A71 transmission is most commonly through fecal-oral and/or oral-oral routes because viruses can be easily detected in oral secretions and feces^2^,^9^. Moreover, it is assumed that the virus mainly enters the host via some part of the orodigestive tract but so far no portal of entry has been confirmed. It was postulated that virus could use the palatine tonsil as an entry portal, based on the localization of viral antigens and RNA within tonsillar crypt squamous epithelium^10^ that strongly suggests infection of these cells. Thus, EV-A71 demonstrates squamous epitheliotropism i.e. has a predilection for squamous cells, in the palatine tonsil. Squamous epitheliotropism in a hamster model^11^ and a transgenic mouse model^12^ has also been demonstrated since squamous cells in the epidermis (keratinocytes) and oral cavity squamous mucosa showed evidence of viral infection. In addition, hamster esophageal squamous mucosa was also found to be infected. Although virus can be readily isolated from mouth ulcers and skin lesions^3^,^13^,^14^,^15^,^16^,^17^, there have been very few pathological studies on infected human skin and oral cavity tissues, and hence no available evidence that squamous cells in these organs are susceptible to infection^10^.

We hypothesize that squamous cells in the epidermis and oral cavity are also susceptible to infection and represent important viral replication sites that contribute significantly to oral and cutaneous virus shedding and viremia. In this study we first investigated if EV-A71 was able to infect human epidermal and oral mucosa squamous cells and perhaps other cell types found in organotypic cultures derived from prepuce and lip tissues. We then studied viral growth characteristics using human primary epidermal squamous cell cultures. Our results strongly suggest that EV-A71 can readily infect human epidermal keratinocytes and oral mucosa squamous cells, thus confirming viral squamous epitheliotropism. Our results show that squamous epitheliotropism play a significant role in oral and cutaneous viral shedding leading to person-to-person viral transmission. As viral replication sites contribute to viremia, squamous epitheliotropism may also play an important role in neuroinvasion, which may be associated with higher viremia.

## Results

### Infection of human skin and oral mucosa organotypic cultures

Tissue morphology assessment of skin organotypic cultures by light microscopy at days 0, 2, 4 and 6 showed day 0 and day 2 tissues to be largely intact. Day 4 tissues showed focal or spotty epidermal cell necrosis and nuclear pyknosis whereas, at day 6, many of the squamous cells from the superficial epidermis started to detach with only suprabasal and basal cells remaining attached to the basement membrane. The dermis and skin appendages appeared normal up to 6 days of culture. A standard cell viability assay using the Celltiter 96^®^ aqueous one solution (Promega, Madison, USA) that measures the reduction of a proprietary MTS tetrazolium compound, estimated the relative cell viability of the skin organotypic cultures at days 2, 4 and 6 to be 88%, 62% and 50% (data not shown), respectively (Day 0 being 100% viability). These results correlated well with light microscopic findings.

**Figure 1:**
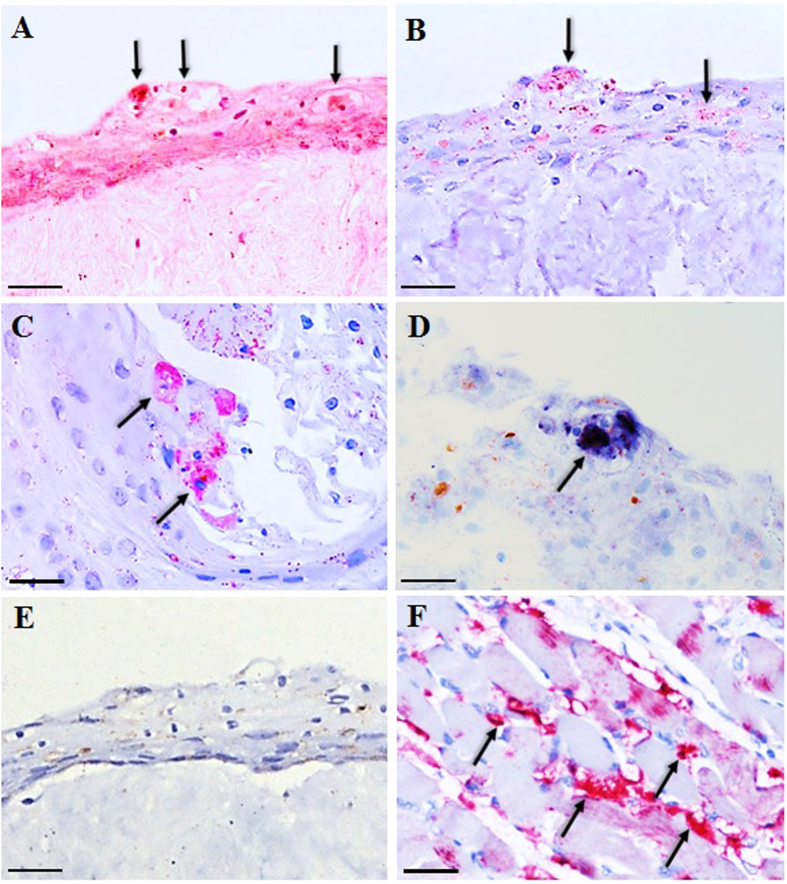
Pathological findings in EV-A71-infected organotypic culture epidermal squamous cells. At 2 days post-infection (dpi), prepuce epidermal squamous cells showed focal necrosis and vacuolated cytoplasm (**A**, arrows) and localization of viral antigens in the same lesion (**B**, arrows) and antigens (**C**, arrows) and viral RNA in other lesions (**D**, arrow). A negative control for the immunohistochemistry procedure that uses anti-Japanese encephalitis virus instead of anti-EV-A71 antibodies is shown in E (same lesion as in A and (**B**). A positive tissue control using EV-A71-infected hamster skeletal muscle is shown in (**F**, arrows). Stains: Hematoxylin and eosin (**A**), immunohistochemistry with permanent red chromogen/hematoxylin (**B**,**C**,**E**,**F**), and *in situ* hybridization with nitroblue tetrazolium/5-bromo-4-chloro-3-indolyl phosphate/hematoxylin (**D**). Original magnification: 20x objective (**A**,**B**,**E**), 40x objective (**C**,**D**,**F**). Scale bars: 30 μm (**A**,**B**,**E**), 15 μm (**C**,**D**,**F**).

**Figure 2:**
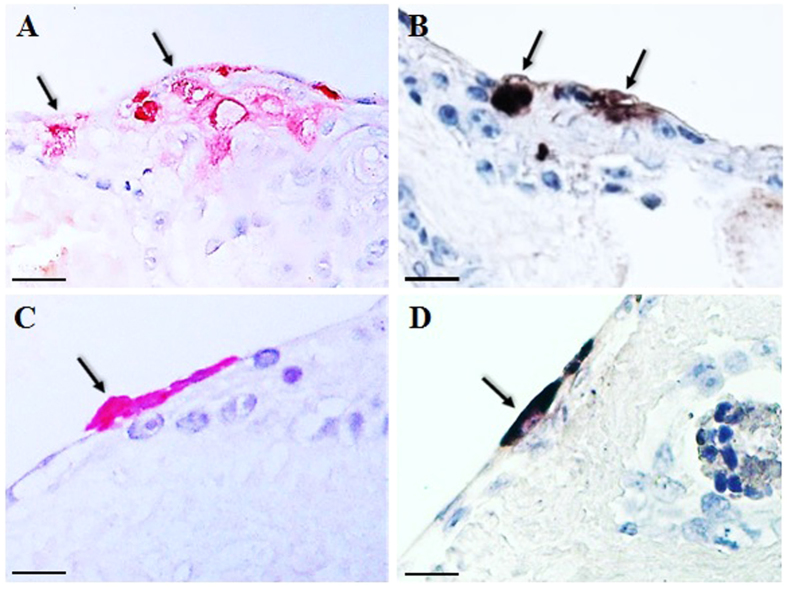
Pathological findings in EV-A71-infected lip epidermal and oral mucosa squamous cells. At 3 days post-infection, lip epidermal squamous lesions demonstrated viral antigens (**A**, arrows) and viral RNA (**B**, arrows) in the same lesion. Similarly, viral antigens (**C**, arrow) and viral RNA (**D**, arrow) were detected in the same infected superficial oral squamous mucosal lesion. Stains: Immunohistochemistry with permanent red chromogen/hematoxylin (**A**,**C**), and *in situ* hybridization with nitroblue tetrazolium/5-bromo-4-chloro-3-indolyl phosphate/hematoxylin (**B**,**D**). Original magnification: 40x objective (**A**,**B**), 60X objective (**C**,**D**). Scale bars: 15 μm (**A**,**B**), 10 μm (**C**,**D**).

Following EV-A71 infection, squamous cells at 2 dpi appeared degenerate and were characterized by vacuolation and nuclear shrinkage (Fig. 1A). Focal EV-A71 infection was detected by immunohistochemistry (IHC) and *in situ* hybridization (ISH) that localized viral antigens and RNA, respectively, only in squamous cell cytoplasm in organotypic cultures of prepuce (Fig. 1B–D) and lip epidermis (Fig. 2A,B), and lip oral mucosa (Fig. 2C,D). EV-A71-infected squamous cells in the prepuce and lip skin organotypic cultures were mostly found below the topmost corneal layer. Infected squamous cells in the oral mucosa could also be found in the most superficial layers. Table 1 summarizes the IHC and ISH findings in these tissues. EV-A71 infection of prepuce epidermis as demonstrated by IHC, averaged about 71% at 2 dpi, 64% at 4 dpi, and 36% at 6 dpi, with an overall mean of 57%. In lip epidermis and/or lip oral mucosa, infection was about 15% at 1 dpi, 42% at 3 dpi, and 35% at 5 dpi, with an overall mean of 30%. Overall, the percentage of ISH-positive fragments was lower than IHC (Table 1). Dermal connective tissues, blood vessels and other tissues were negative for viral antigens/RNA. Positive controls showed strong signals for viral antigens/RNA in infected hamster skeletal muscle tissues (Fig. 1F) but were undetectable in the negative controls (Fig. 1E).

**Figure 3:**
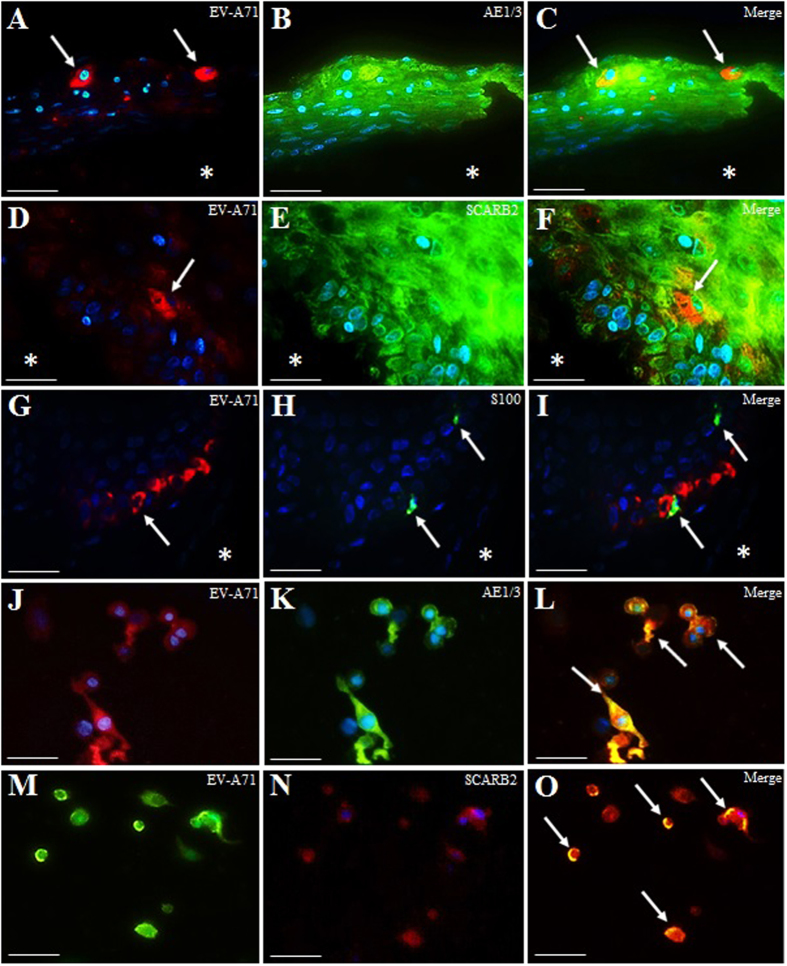
Double immunofluorescence staining of EV-A71-infected human prepuce and lip epidermis, and primary epidermal keratinocytes. Viral antigens were found in cytoplasm of prepuce epidermal keratinocytes (**A**,**C**, arrows) which were uniformly cytokeratin AE1/3 positive (**B**). Viral antigens in cytoplasm of prepuce epidermal keratinocytes (**D**,**F**, arrows) in uniformly SCARB2-protein positive keratinocytes (**E**). Both AE1/3 and SCARB2 (**B**,**C**,**E**,**F**) were negative in the dermal layers (*). S100 protein-positive Langerhans cells (**H**, arrows) were found adjacent to keratinocytes with viral antigens (**G**,**I**, arrows). There was no apparent co-localization of viral antigens in Langerhans cells. The dermal layer (*) is negative for both S100 and viral antigens (**H**,**I**). Viral antigens were found in AE1/3-positive primary epidermal keratinocytes (**J**–**L**, arrows) and SCARB2-positive primary keratinocytes (**M–O**, arrows). Stains: Immunofluorescence staining with permanent red chromogen (**A**,**D**,**G**,**J**), anti-human cytokeratin AE1/3/IgG conjugated with Alexa Fluor 488 (**B**,**K**), SCARB2/IgG conjugated with Alexa Fluor 488 (**E**) and Alexa Fluor 594 (**N**), S100/IgG conjugated with Alexa Fluor 488 (**H**), and DAPI (4′,6-diamidino-2-phenylindole, dihydrocholoride). Original magnification: 60x objective (**A**–**O**). Scale bars: 10 μm (**A**–**O**).

Double IF using the epithelial marker AE1/3 and anti-EV-A71 antibody confirmed viral antigen localization in the cytoplasm of prepuce epidermal keratinocytes (Fig. 3A–C). Similarly, like AE1/3, the well-known EV-A71 receptor, scavenger receptor class B member 2 (SCARB2) was also found to be positive in all keratinocytes, and co-localized with viral antigens (Fig. 3E,F). Although S100 protein-positive Langerhans cells (epidermal dendritic cells) were detectable by IF, we did not find convincing evidence of viral antigens in Langerhans cells using double IF to detect anti-S100 protein and viral antigens (Fig. 3G–I). Very rarely, a Langerhans cell may be seen adjacent to an infected cell.

### EV-A71 replication in prepuce skin organotypic culture

**Figure 4:**
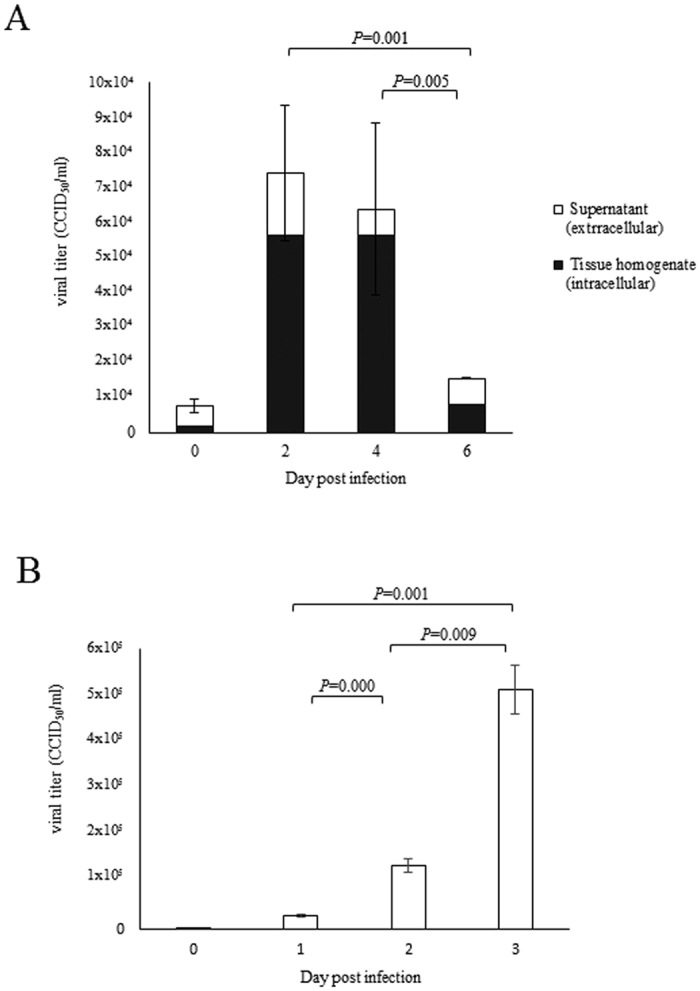
Virus replication in (**A**) EV-A71-infected human prepuce skin at 2, 4 and 6 days post-infection (dpi), and (**B**) primary epidermal keratinocytes at 1, 2, 3 dpi. Viral titers are expressed as CCID_50_/ml. Combined viral titers from organotypic tissue homogenates (intracellular) and supernatant (extracellular) were statistically significant between 2 and 6 dpi (*P* = 0.001) and between 4 and 6 dpi (*P* = 0.005), respectively. Supernatant viral titers from primary epidermal keratinocytes (**B**) between all time points were significantly significant (*P* < 0.05).

Viral titers from infected prepuce skin organotypic cultures are shown in Fig. 4A. The combined viral titers from supernatant (extracellular titer) and tissue homogenates (intracellular titer) peaked at 2 dpi. The combined viral titer at 2 dpi of 7 × 10^4^ CCID_50_ (50% cell culture infective dose) was significantly higher (*P* = 0.001) than at 6 dpi (1 × 10^4^ CCID_50_). Similarly, viral titers at 4 dpi (6 × 10^4^ CCID_50_) were also significantly higher (*P* = 0.005) than at 6 dpi. Overall, corresponding viral titers derived from tissue homogenates collected from the same wells were approximately 10-fold higher than titers from supernatant at all time points.

### EV-A71 replication in primary epidermal keratinocyte culture

**Figure 5:**
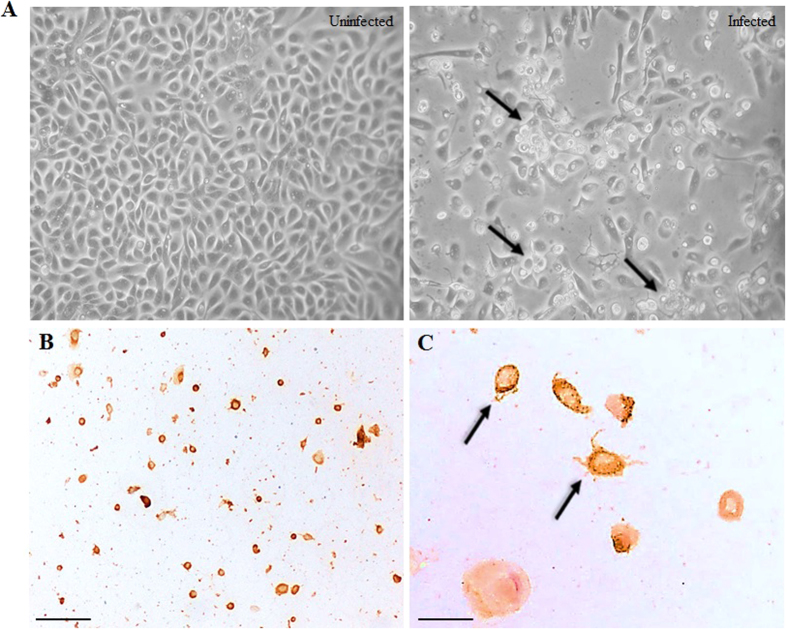
Infection of primary epidermal keratinocytes. Cytopathic effect (**A**, arrows) at days post-infection. Infected keratinocytes stained by immunohistochemistry with 3, 3′ diaminobenzidinetetrahydrochloride chromogen/hematoxylin at low (**B**) and high magnifications (**C**, arrows). Original magnification: 10x objective (**B**), 40x objective (**C**). Scale bars: 50 μm (**B**), 15 μm (**C**).

Susceptibility of human squamous EV-A71 infection was confirmed using a primary prepuce epidermis keratinocyte culture. Infection of keratinocyte monolayers at an MOI of 0.05 (10^5^ CCID_50_) from 2 dpi onwards showed cytopathic effects (CPE), consisting of swelling and rounding up of cells (Fig. 5A). About 90% of cells showed CPE at 3 dpi, and viral antigens were demonstrated in these cells (Fig. 5B,C). Uninfected primary keratinocyte controls showed no CPE. The cell monolayers were confirmed to be keratinocytes that stained strongly positive for AE1/3 IHC (data not shown). Moreover, double IF (AE1/3 and viral antigen staining) confirmed keratinocyte infection (Fig. 3J–L), and infected keratinocytes also strongly expressed SCARB2 receptors (Fig. 3M–O).

Virus titration from the supernatant showed significant increase of titers for all time points (Fig. 4B). The highest titer of 5 × 10^5^ CCID_50_ at 3 dpi was about 25-fold higher than 2 × 10^4^ CCID_50_ at 1 dpi (*P* = 0.001). The average supernatant viral titers from primary epidermal keratinocytes at 2 dpi (1 × 10^5^ CCID_50_) was about 10-fold higher than supernatant from infected organotypic culture at 2 dpi (1 × 10^4^ CCID_50_) (Fig. 4). At 3 dpi (5 × 10^5^ CCID_50_), the titer was about 70-fold higher than organotypic tissue supernatant at 4 dpi (7 × 10^3^ CCID_50_). Thus, overall supernatant viral titers from primary epidermal keratinocytes were higher than from organotypic cultures.

## Discussion

Based on clinical observations and viral culture results, the skin in various parts of the body especially in the hand, foot, lips, buttock, and oral mucosa are well known to be infected but the cellular target/s of virus in these tissues have remained unknown. In our study, squamous cells derived from human prepuce and lip epidermis, and lip oral mucosa were found to be infected by EV-A71. Focal squamous cell degeneration and necrosis was associated with EV-A71 antigens/RNA as demonstrated by specific IHC and ISH assays within the cytoplasm of epidermal keratinocytes and oral mucosa squamous cells (Figs 1 and 2). As far as we are aware, this is the first time that EV-A71 squamous epitheliotropism has been reported in human tissues. Due to a lack of suitable naturally-infected human tissues for investigation, very little data has been published except in one available skin sample in which viral antigens were not detected^10^. Nevertheless, the authors found palatine tonsil crypt squamous epithelia to be infected by EV-A71. Further investigations using human autopsy or biopsy tissues are needed to confirm EV-A71 squamous epitheliotropism.

In EV-A71-infected hamsters, multiple, focal inflammatory skin lesions were found on footpads/paws, lips and other parts of the skin following oral infection^11^. Viral antigens/RNA were also localized within the cytoplasm of epidermal squamous cells found in these areas, as well as squamous cells of the oral and oesophageal mucosa. Similarly, a SCARB2 transgenic mouse model has also convincingly demonstrated viral antigens in squamous cells in the oral mucosa and in the skin on limbs^12^. In human primary epidermal keratinocytes, we found viral antigens in AE1/3-positive keratinocytes (Fig. 3L) confirming squamous epitheliotropism. All keratinocytes were also SCARB2-positive (Fig. 3O) confirming the ubiquitous distribution of SCARB2 in most cells and tissues^18^,^19^,^20^. Other HFMD-causing, SCARB2-associated enteroviruses such as coxsackievirus A7 (CVA-7), CVA-14 and CVA-16^21^ may likewise infect squamous cells but so far there is no evidence for this. HFMD-causing but non-SCARB2-associated CVA-6 viral antigens have been demonstrated in keratinocytes found around a skin lesion of epidermal necrosis and vesicle formation in a naturally-infected human case^22^. Hence, other viral receptors in squamous cells may facilitate CVA-6 entry. In other viral infections that cause skin vesicles and rashes such as herpes simplex and varicella zoster, viral antigens have been demonstrated within squamous cells^22^,^23^,^24^,^25^,^26^.

In EV-A71-infected prepuce skin organotypic cultures, the highest viral titers were observed at 2 dpi which significantly dropped after 4 dpi suggesting that virus was probably most actively replicating from 2 dpi to 4 dpi. The reasons for the reduction in viral titers at 4 dpi may in part be related to reducing tissue viability after 4 days of culture as evidenced by light microscopy and the Celltiter 96^®^ assay (Promega, Madison, USA). EV-A71 replication in primary epidermal keratinocytes increased from 1 dpi to reach the highest titers at 3 dpi, confirming robust growth in keratinocytes. Up to 60% of skin vesicle fluid/swabs from HFMD patients were found to be positive by viral culture^3^,^13^,^15^ but as far as we are aware, there are no published data on viral titrations from these samples. Moreover, PCR has been increasingly used for viral detection and reported as either positive or negative. Our data provides an indication that viral replication in epidermal keratinocytes is very active and this may also be the case *in vivo*. Hence, virus shedding from skin vesicles could be sufficient to facilitate significant person-to-person transmission by the cutaneous-to-oral route. We speculate that cutaneous-to-oral transmission probably occurs more readily than oral-to-cutaneous route because the most superficial epidermal corneal layer is well known to resist infection. Theoretically, transmission via an oral-to-cutaneous route may still be possible if a sufficient viral dose is delivered onto the skin followed by direct infection of the more superficial layers of the epidermis^2^,^27^. However, in the prepuce and lip skin organotypic cultures, infected squamous cells were mostly below the superficial corneal layer. However, skin breaches in the form of cuts or abrasions may predispose to infection. Conversely, devoid of a corneal layer, oral squamous mucosa would probably be more readily infected directly by virus. In fact, we were able to demonstrate that the most superficial squamous mucosal cells could be infected (Fig. 2C,D). However, from our findings in the organotypic cultures, it was not possible to determine with certainty if virus is more likely to infect from the apical or basal surfaces of squamous cells.

Apart from tonsillar crypt epithelium^10^, oral mucosa could be another major primary replication site for EV-A71^28^, and indeed may be a portal for viral entry in the body as well. Having gained entry, viremia is likely to spread virus to the epidermis in the feet, buttocks and other areas, which are less likely to be exposed to direct viral contact. In this case, the skin lesions would represent secondary viral replication sites. Thus, the data provide evidence that squamous epitheliotropism plays a crucial role in the viral replication and dissemination in the body. Since skin lesions are more commonly observed in the perioral skin, hands and feet, it is possible that viral growth in epidermal keratinocytes in these areas may be higher than in the prepuce and other parts of the body. The factors that could explain EV-A71 predilection for skin in these parts of the body have to be further investigated.

We did not detect viral antigens/RNA in dermal fibroblasts, blood vessels, peripheral nerves or other cells/tissues suggesting that these cells/tissues were not susceptible to the infection. Epidermal dendritic cells, Langerhans cells (LCs), were not shown to have viral antigens in our organotypic skin cultures but this possibility should be investigated more thoroughly. In *in vitro* experiments, EV-A71 has been shown to infect dendritic cells, which are professional antigen-presenting cells that prime T lymphocytes^29^,^30^. In our study, no viral antigens were detected in LCs for various possible reasons, including a reduction in the viability of LCs in skin organotypic cultures and a relatively short period of culture that precludes phagocytosis of viral antigens/virus. Further experiments are needed to investigate the role of LCs in EV-A71 skin infection.

In conclusion, evidence from our infection experiments using human skin and oral mucosa organotypic cultures and primary squamous cell cultures confirmed squamous epitheliotropism of EV-A71. Our results show that human squamous cells/keratinocytes are important viral targets and represent important primary and/or secondary viral replication sites that may contribute significantly to viremia and person-to-person transmission. Moreover, judging from the very similar clinical manifestations, we predict that all enteroviruses that cause HFMD are squamoepitheliotropic.

## Materials and Methods

### Virus stock preparation

Vero cells were grown in Dulbecco’s modified Eagle’s growth medium (DMEM) supplemented with 10% fetal bovine serum (FBS). A human EV-A71 strain isolated from a child with HMFD, designated as A104 (genogroup B4; Genbank No.: AF376067), was used throughout this study. To prepare the stock, virus was inoculated onto Vero cell monolayers in DMEM with 2% FBS at a multiplicity of infection (MOI) of 0.01, and virus titers were determined by a standard microtitration assay as described previously^31^, and calculated using the Karber method^32^.

### Human tissue organotypic culture

The study was approved by the Medical Ethics Committee, University of Malaya Medical Centre in accordance with ICH Harmonised Tripartite Guidelines for Good Clinical Practice (Registration No. 920.1). All donors have given written informed consent prior to sample collection. Prepuce and lip skin and lip oral mucosa tissues were obtained from circumcisions and cleft-lip repair surgeries, respectively, in children aged 3 months to 9 years. These biopsy samples were cultured as described previously with minor modifications^23^,^33^. Briefly, the specimens were immediately transferred to the lab in a sterile tube containing cold phosphate buffered saline (PBS, pH 7.4). Next, the specimens were washed with PBS containing penicillin and streptomycin (20 μl/ml) (Sigma-Aldrich, St. Louis, USA) and fungizone (10 μl/ml) (Sigma-Aldrich, St. Louis, USA), before further disinfection in 70% ethanol for a few seconds. Samples were then washed twice in PBS before careful dissection to obtain approximately 1 mm × 3 mm tissue fragments. Eight fragments per well were placed on a transwell insert membrane with a 0.4 μm pore size (BD Falcon^TM^, New York, USA) at air-liquid interface in 10% DMEM, at 37 °C for experiments.

### Cell Proliferation Assay

A cell proliferation assay using Celltiter 96^®^ aqueous one solution (Promega, Madison, USA), a standard and sensitive colorimetric quantification based on the reduction of a proprietary MTS tetrazolium compound by NAD(P)H-dependent dehydrogenase enzymes in metabolically active cells, was performed to assess general skin organotypic culture viability. Prepuce skin from 4 individual patients cultured under the usual conditions as described above were harvested on days 0, 2, 4 and 6, before transfer it to a 96-well plate for incubation with Celltiter aqueous solution for 4 hours at 37 °C in the dark, according to manufacturer’s protocol. After incubation, the absorption spectrum of the solution was measured at a wavelength of 490 nm with microplate reader (BioTek, Winooski, USA). Since for each time point, 4 tissue fragments from each patient were harvested, a total of 64 fragments were assessed for viability.

### Infection of human organotypic cultures

Seven sets of human prepuce skin organotypic cultures, derived from 7 patients, were grown in 6-well plates, and separately harvested for study at 2, 4 and 6 dpi. Each set consisting of 3 wells (10 tissue fragments/well) were infected with 2 × 10^6^ CCID_50_ of A104 virus per well. The plates were then shaker-incubated at 37 °C for 2 hours to allow for virus attachment and entry. The specimens were then thoroughly washed 3 times with PBS containing antibiotics, before transfer onto transwell insert membranes placed in another set of 6-well plates each containing 3.2 ml/well of new medium. The medium was maintained unchanged for the whole experiment. To determine viral titers at 0 dpi, 100 μl of the medium and 2 tissue fragments were collected from each set of organotypic cultures very soon after the new medium was added. At 2, 4 and 6 dpi, respectively, 6 tissue fragments and supernatant per well were collected for viral titration. Tissue fragments were washed with PBS 3 times, air dried for about 10 minutes at room temperature (RT) before weighing and freezing at −80 °C for later viral titration. Briefly, tissues were homogenized in PBS to obtain 10% (wt/vol) suspensions and virus titers were detected using microtitration assay in Vero cells as described previously^31^.

The remaining 2 fragments in each well were harvested for light microscopy, IHC and ISH at 2, 4 and 6 dpi, respectively. For uninfected control tissues, duplicate cultured specimens from all 7 patients prepared in the same way were harvested for IHC and ISH at day 2, 4 and 6 (n = 8 each).

Lip skin and/or oral mucosa tissues from 4 patients (8 tissue fragments per patient, 8 fragments/well) were infected and shaker-incubated as before with the same viral dose. The medium was changed twice a week for the duration of the experiment. Two tissue fragments were harvested from each well at 1, 3 and, 5 dpi, respectively, for light microscopy, IHC and ISH. Uninfected controls, were prepared as before. Due to the limited size and availability of samples, virus titration from infected lip skin/oral mucosa tissues was not done.

### Primary epidermal keratinocyte monolayer culture

Human prepuce skin biopsies were cut into fragments of about 3 × 3 mm and placed in a 30 mm dish containing 3–4 ml of PBS with antibiotics and 2 U/ml of Dispase (Sigma-Aldrich, St. Louis, USA) overnight at 4 °C. The epidermal layer was then carefully peeled off using sterile forceps, and immediately washed with PBS/antibiotics before further digestion with 0.05% trypsin for 30 minutes at 37 °C to obtain keratinocyte suspensions. After washing with hepes buffered saline (Lonza, Walkersville, USA) and centrifugation at 176 × g for 5 minutes at RT, the cells were cultured in a keratinocyte growth medium (Lonza, Walkersville, USA), in T25 flasks at 37 °C for 2–3 weeks, changing medium every 2 days.

### Infection of primary epidermal keratinocytes

After the 1^st^ passage, keratinocytes were seeded into 12-well plates and cultured at 37 °C for 2 days. When 70–80% confluence was achieved, the cells were incubated with A104 virus at a MOI of 0.05 (10^5^ CCID_50_) at 37 °C for 2 hours for virus attachment and entry. The cells were then thoroughly washed with PBS containing antibiotics 3 times, new medium added and culture maintained up to 3 days with no further medium change. Supernatant was harvested from each 4 well replicates for virus titration at 0, 1, 2 and 3 dpi, respectively, as described.

### Light microscopy

Two organotypic culture tissue fragments per well were harvested from infected wells (2, 4, 6 dpi) and uninfected wells (0, 2, 4, 6 days of culture), and immediately fixed in 10% neutral-buffered formalin before routine processing and paraffin embedding. Similarly, lip and/or oral mucosa tissues were harvested at 1, 3, 5 dpi and processed as described. Tissue blocks were microtomed at 4 μm and tissue sections were stained with routine hematoxylin and eosin for light microscopy.

### IHC

A modified IHC technique was used to localize EV-A71 antigens in organotypic culture tissues^11^. Briefly, 4 μm deparaffinised tissue sections were incubated with primary monoclonal mouse anti-EV-A71 antibody (Light Diagnostics, Livingston, UK) overnight at 4 °C after antigen retrieval and normal serum blocking. Secondary antibody goat anti-mouse IgG conjugated with enzyme alkaline phosphatase (Santa Cruz Biotechnology, Dallas, USA) was applied to the sections for 30 minutes at RT before colour development with Liquid Permanent Red chromogen (Dako, Santa Clara, USA). Tissue sections were counterstained with hematoxylin and mounted with DPX mounting media (Sigma-Aldrich, St. Louis, USA). A total of 42 infected tissue fragments from prepuce and 56 fragments from lip skin and oral mucosa were examined. For negative IHC controls, mouse isotype control IgG1 or Tris buffered saline (TBS), or anti-Japanese encephalitis virus antibodies was used instead of the primary antibody. Known EV-A71-infected mouse and hamster tissues^11^,^31^ were used as positive controls. IHC specificity testing was also done on corresponding uninfected cultured tissues as negative controls.

IHC using a mouse monoclonal anti-human cytokeratin AE1/3 (Dako, Santa Clara, USA) was also performed to confirm that keratinocyte monolayers were grown from human prepuce. Briefly, cells were incubated with primary antibody overnight at 4 °C followed by secondary antibody-horseradish peroxidase (Dako Real Envision, Produktionsvej, Denmark) for 30 minutes at RT. The substrate chromogen 3,3′ diaminobenzidine tetrahydrochloride (Dako, Produktionsvej, Denmark) was then applied, followed by hematoxylin counterstaining and mounting. For negative IHC controls, the primary antibody was replaced by mouse isotype control IgG1 or TBS.

IHC to detect viral antigens in infected primary keratinocytes was done at 3 dpi after serial washing with PBS and fixation in absolute methanol. The IHC procedure was similar to that for organotypic culture tissues except for incubation with secondary antibody-horseradish peroxidase for 30 minutes at RT (Dako Real Envision, Produktionsvej, Denmark) and that use of a different substrate chromogen 3,3′ diaminobenzidine tetrahydrochloride (Dako, Produktionsvej, Denmark) before hematoxylin counterstaining and mounting. EV-A71-infected Vero cells were used as positive controls and uninfected primary keratinocytes were used as negative controls.

### Double immunofluorescence (IF)

To demonstrate co-localization of EV-A71 antigens in cells with SCARB2, an important viral receptor, double IF was performed on selected organotypic prepuce (n = 8) and lip (n = 2) skin, and primary keratinocyte cultures (n = 4) confirmed earlier by IHC to have viral antigens. Anti-EV-A71 antibody (Light Diagnostics, Livingston, UK) was applied onto the tissue sections and incubated overnight at 4 °C followed by secondary antibody goat anti-mouse IgG conjugated with enzyme alkaline phosphatase (Santa Cruz Biotechnology, Dallas, USA) for 30 minutes at RT in the dark before colour development with Liquid Permanent Red chromogen (Dako, Santa Clara, USA). Next, a polyclonal rabbit anti-LIMPII/lgp85 antibody (GeneTex, Irvine, USA) was applied and incubated overnight at 4 °C followed by goat anti-rabbit IgG conjugated with Alexa Fluor 488 (Life Technologies, Carlsbad, USA) for 30 minutes at RT in the dark. The same anti-EV-A71 antibody and a polyclonal rabbit anti-LIMPII/lgp85 (SCARB2) antibody (GeneTex, Irvine, USA) which has been used extensively to detect SCARB2 were applied together onto the primary keratinocytes and incubated overnight at 4 °C followed by goat anti-mouse IgG conjugated with Alexa Fluor 488 (Life Technologies, Carlsbad, USA) and goat anti-rabbit IgG conjugated with Alexa Fluor 594 for 30 minutes at RT in the dark. Tissues/cells were then counterstained with DAPI (4′,6-diamidino-2-phenylindole, dihydrocholoride) (Molecular Probes, Eugene, USA) for 1 minute followed by TBS washes. Slides were viewed with an inverted research microscope ECLIPSE Ti (Nikon, Tokyo, Japan).

Localization of viral antigens specifically within prepuce epidermis (n = 8) and primary keratinocytes (n = 4) was demonstrated by the same IF procedure using monoclonal mouse anti-human cytokeratin AE1/3 (Dako, Santa Clara, USA) instead of anti-LIMPII/lgp85 antibody, and goat anti-mouse IgG conjugated with Alexa Fluor 488 was used. To detect viral antigen co-localization in skin Langerhans cells (n = 16), the same IF procedure was followed except that an anti-S100 antibody (Abcam, Cambridge, UK), a well-known and reliable cell marker for Langerhans cells was applied instead of anti-LIMPII/lgp85 antibody, and goat anti-rabbit IgG conjugated with Alexa Fluor 488 was used. EV-A71-infected hamster tissues and Vero cells were used as positive controls, and uninfected keratinocytes as negative controls. Human rhabdomyosarcoma cells were used as positive controls for SCARB2 detection.

### ISH

A ISH procedure as described previously was used to detect EV-A71 RNA^11^. Briefly, tissue sections of prepuce skin (n = 41) and lip skin and/oral mucosa (n = 47) were pre-treated with 0.2 N HCL and proteinase K (100 μg/ml) digestion for 20 minutes at 37 °C, before hybridization with 50 μl of hybridization solution at 95 °C for 10 minutes, followed by incubation for 16 hours at 42 °C in a moist chamber^34^. Hybridization was detected using anti-DIG-Alkaline-Phosphatase-conjugate (Roche, Mannheim, Germany) followed by nitroblue tetrazolium/5-bromo-4-chloro-3-indolyl phosphate (Roche, Mannheim, Germany) substrate for color development. For negative ISH controls, duplicate assays that omitted probes, and uninfected human skin cultured tissues were used. EV-A71-infected hamster tissues were used as positive controls^11^.

### Statistics

To determine statistical significance between viral titers, one-way anova was performed followed by repeated t-test using the software IBM SPSS Statistics version 23. The results were expressed as mean ± standard deviation. A *p* value of <0.05 was considered significant.

## Additional Information

**How to cite this article**: Phyu, W. K. *et al*. Squamous epitheliotropism of Enterovirus A71 in human epidermis and oral mucosa. *Sci. Rep.* **7**, 45069; doi: 10.1038/srep45069 (2017).

**Publisher's note:** Springer Nature remains neutral with regard to jurisdictional claims in published maps and institutional affiliations.

## Figures and Tables

**Table 1:** Immunohistochemsitry (IHC) and *in situ* hybridization (ISH) findings in human prepuce and lip organotypic cultures.

Organotypic culture	IHC results (no. of positive fragments/total fragments)			ISH results (no. of positive fragments/total fragments)		
	2 DPI	4 DPI	6 DPI	2 DPI	4 DPI	6 DPI
**Prepuce epidermis**						
Case 1	1/2	1/2	0/2	0/2	1/2	0/2
Case 2	1/2	1/2	0/2	0/2	0/2	0/2
Case 3	2/2	2/2	0/2	2/2	0/2	0/2
Case 4	2/2	2/2	1/2	0/1	0/2	0/2
Case 5	1/2	1/2	2/2	1/2	0/2	0/2
Case 6	2/2	1/2	1/2	2/2	0/2	2/2
Case 7	1/2	1/2	1/2	2/2	1/2	1/2
Total positive fragments/total fragments	10/14 (71%)	9/14 (64%)	5/14 (36%)	7/13 (54%)	2/14 (14%)	3/14 (21%)
	**1 DPI**	**3 DPI**	**5 DPI**	**1 DPI**	**3 DPI**	**5 DPI**
**Lip epidermis and oral mucosa**						
Case 1	1/2	3/4	1/1	0/2	1/3	0/1
Case 2	0/6	1/5	2/8	0/6	1/5	0/4
Case 3	0/4	2/4	2/4	0/4	0/4	0/4
Case 4	2/8	2/6	1/4	0/4	1/6	0/4
Total positive fragments/total fragments	3/20 (15%)	8/19 (42%)	6/17 (35%)	0/16 (0%)	3/18 (17%)	0/13 (0%)

DPI = Days post-infection.

For IHC or ISH, 1 positive cell in each fragment is sufficient for the fragment to be counted as a positive result.
